# A Phagocytic Route for Uptake of Double-Stranded RNA in RNAi

**DOI:** 10.1371/journal.pone.0019087

**Published:** 2011-04-29

**Authors:** João J. E. Rocha, Viktor I. Korolchuk, Iain M. Robinson, Cahir J. O'Kane

**Affiliations:** 1 Department of Genetics, University of Cambridge, Cambridge, United Kingdom; 2 Peninsula College of Medicine and Dentistry, University of Exeter, Plymouth, United Kingdom; Institut Pasteur, France

## Abstract

RNA interference (RNAi) has a range of physiological functions including as a defence mechanism against viruses. To protect uninfected cells in a multicellular organism, not only a cell-autonomous RNAi response is required but also a systemic one. However, the route of RNA spread in systemic RNAi remains unclear. Here we show that phagocytosis can be a route for double-stranded RNA uptake. Double-stranded RNA expressed in *Escherichia coli* induces robust RNAi in *Drosophila* S2 cells, with effectiveness comparable to that of naked dsRNA. We could separate this phagocytic uptake route from that for RNAi induced by naked dsRNA. Therefore, phagocytic uptake of dsRNA offers a potential route for systemic spread of RNAi.

## Introduction

RNAi is a type of post-transcriptional gene regulation in which a double-stranded RNA (dsRNA) molecule directs the specific degradation of the target mRNA [1], [2]. At least in some organisms RNAi is not only an intracellular phenomenon, but a systemic one that spreads between cells [3]–[5]. This spread is usually assumed to be mediated by extracellular naked dsRNA [5], and at least two mechanisms for dsRNA uptake have been described, which may not be mutually exclusive. One pathway is a transmembrane channel-mediated uptake mechanism exemplified by the *Caenorhabditis elegans* SID-1 protein [5]. However in some organisms, including *Drosophila*, no *sid* gene orthologs have been found and no general systemic RNAi has been demonstrated [6]. However, *Drosophila* hemocyte-like S2 cells exhibit some features of systemic RNAi, since exogenously added dsRNA can mediate RNAi in them. They take up dsRNA via SID-1-independent endocytic mechanisms that employ the scavenger receptors CI (Sr-CI) and Eater [7], [8]. The situation is however, complicated by the fact that dsRNA uptake mediated by SR-CI and Eater can be separated from RNAi; downregulation of these scavenger receptors can inhibit dsRNA uptake but is reported not to inhibit RNAi [7], [8]. Therefore, it is possible that spread of RNAi in *Drosophila* might use routes other than direct uptake of dsRNA. We hypothesized that phagocytosis could be such an alternative route and tested whether RNAi could be induced by dsRNA-expressing *E. coli* in phagocytic S2 cells.

## Results and Discussion

### dsRNA-expressing *E. coli* induces RNAi in S2 cells

We used *E. coli* as a tool to test whether phagocytosis is a possible route for RNAi. We synthesized several dsRNAs in *E. coli* that lacked the double-strand-specific RNaseIII, using a plasmid vector that harboured two T7 promoters in inverted orientations flanking either a fragment of each gene of interest, or a multiple cloning site of approximately 200 bp with no insertion [9]. T7 RNA polymerase expression was induced using isopropyl β-D-1-thiogalactopyranoside (IPTG).

**Figure 1:**
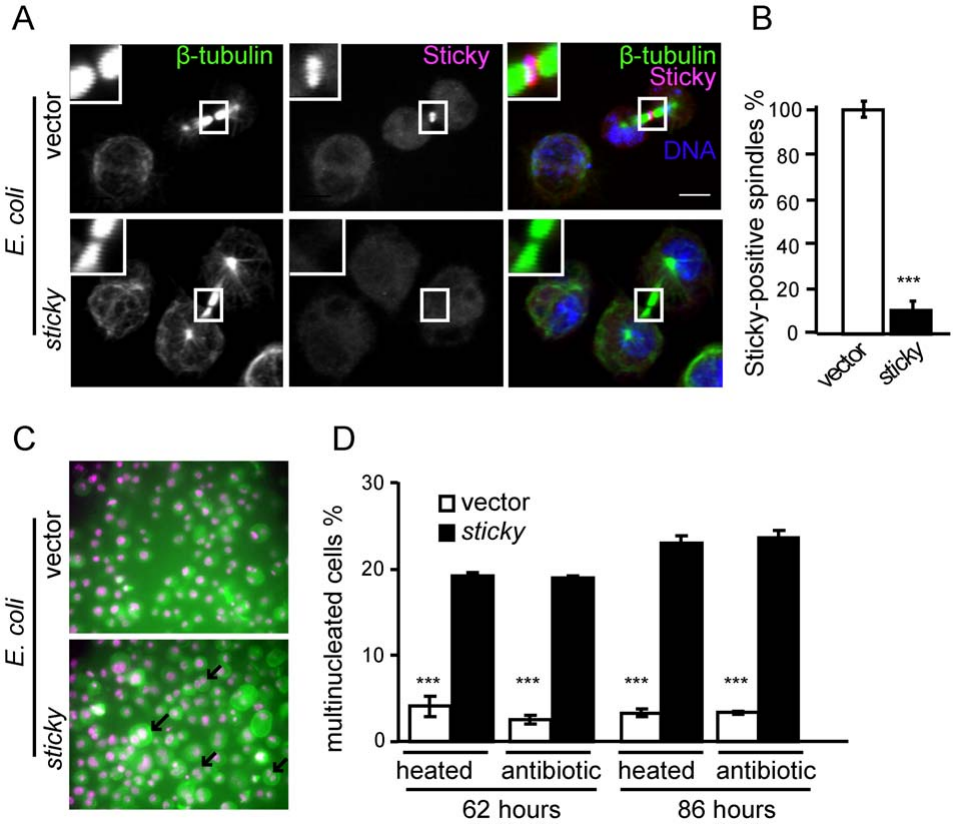
Knockdown of *sticky* expression by dsRNA-expressing *E. coli*. (**A**) S2 cells after 86 hours incubation with *E. coli* cells, stained for β-tubulin, Sticky and DNA (Hoechst-33342). Scale bar, 5 µm. Central spindle figures positive for Sticky were quantified in (**B**). (**C**) Incubation of S2 cells with *E. coli* that expressed *sticky* dsRNA increased the percentage of multinucleated cells in culture. Representative micrographs showing multinucleated S2 cells (e.g. arrows), cultured in the presence of *E. coli* that express *sticky*-dsRNA or carry pPD129.36 vector only (4), and stained with phalloidin (green) and the nuclear dye Hoechst-33342 (magenta). Cells were cultured for 62 or 86 hours in a 10-fold excess of IPTG-treated *E. coli*. To inhibit bacterial growth, *E. coli* cells were either heat-inactivated prior to incubation or cultured in the presence of antibiotics (penicillin and streptomycin). (**D**) Bar graph showing percentage of multinucleated S2 cells in cultures treated as (**C**). Values are averages of four independent experiments, each averaged from 200–500 S2 cells. Error bars indicate standard error of the mean. *** P<0.005; two-tailed t-test.

Initially, we tested the effectiveness of *E. coli* that carried a fragment of *sticky*, a gene that encodes a citron kinase, necessary for completion of cytokinesis [10], in knocking down expression and function of *sticky*. Consistent with a phagocytic uptake route, *E. coli* that expressed *sticky* dsRNA greatly reduced the levels of Sticky protein detected during cytokinesis in S2 cells (Fig. 1A, B). We also observed a characteristic *sticky* mutant phenotype [10]: inhibition of cytokinesis and consequently increased numbers of multinucleate cells (Fig. 1C). Both heat-inactivated and non-inactivated bacteria inhibited cytokinesis, and inhibition increased with longer incubation times (Fig. 1D).

**Figure 2:**
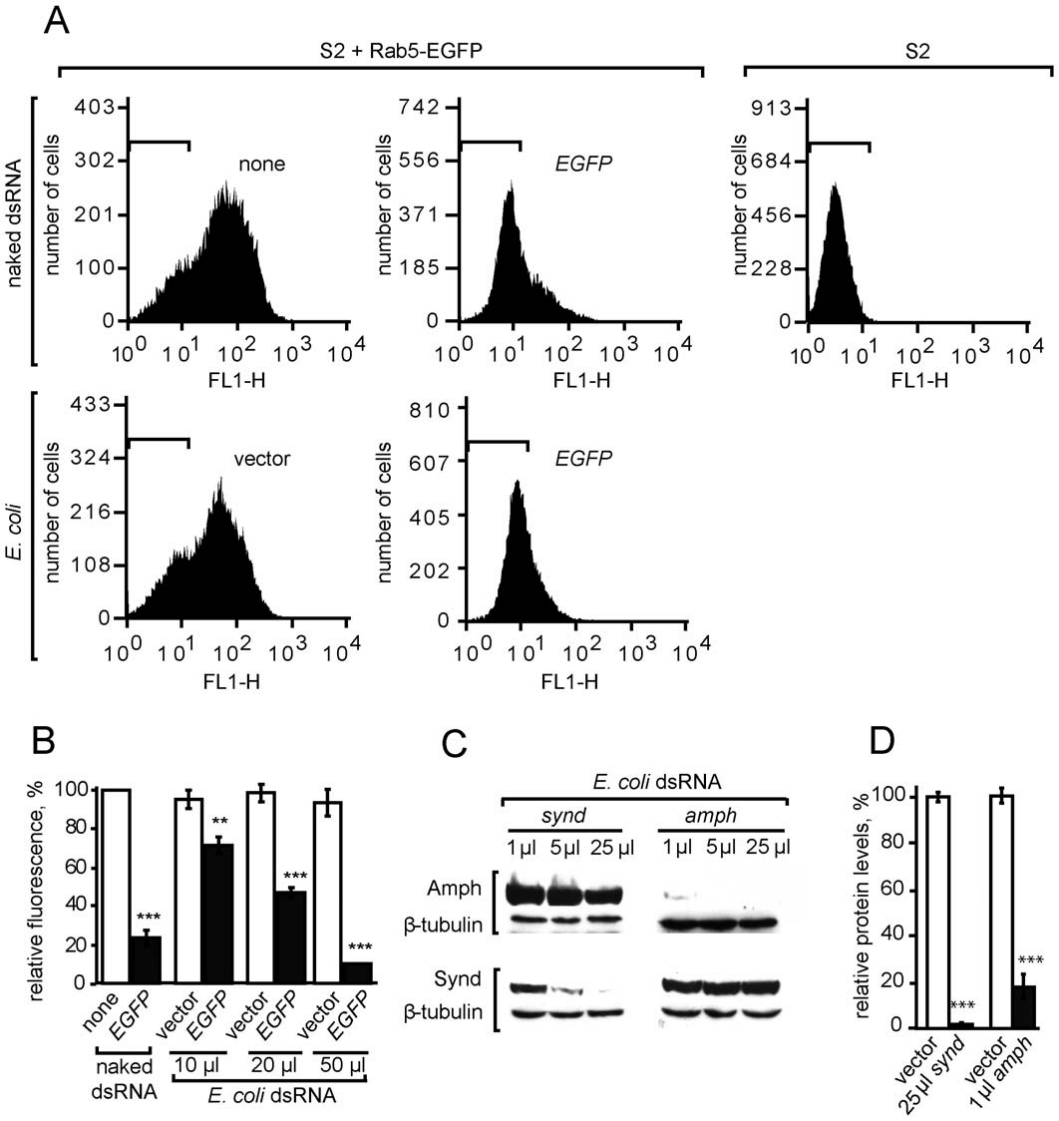
Efficiency and specificity of RNAi induced by dsRNA-expressing *E. coli*. (**A**) Fluorescence loss in Rab5-EGFP-expressing S2 stable cell line due to RNAi induced either by 1 µg of naked EGFP dsRNA or by 50 µl of EGFP dsRNA-expressing *E.coli*. Representative FACS histograms are showing numbers of S2 cells with different fluorescence intensities. Horizontal bars in each graph (approximately 100 to 101) show the fluorescence range of wild-type S2 cells (as in the top right histogram). (**B**) Quantification of EGFP fluorescence levels in Rab5-EGFP-expressing S2 cells, treated with either 1 µg of EGFP dsRNA or varying quantities of *E. coli* (OD600 = 1.2) that express EGFP dsRNA. The graph shows the level of EGFP fluorescence, normalized to cells treated with no RNAi. (**C**) Western blots of amphiphysin, syndapin or β-tubulin in lysates from S2 cells treated for 72 hours with *E. coli* that express *amph* or *synd* dsRNA. (**D**) Bar graph shows levels of amphiphysin and syndapin relative to β-tubulin. Knockdown was quantified in S2 cells treated with *E. coli* that expressed *synd* (25 µl) or *amph* (1 µl) dsRNA; amphiphysin was undetectable after treatment with larger amounts of *E. coli*. Values are averages of three independent experiments. Error bars indicate standard error of the mean. ** P<0.01; *** P<0.005; two-tailed t-test.

To test whether bacterially delivered dsRNA could induce RNAi as effectively as naked dsRNA, we challenged S2 cells that expressed EGFP-Rab5 with *EGFP* dsRNA, via both delivery routes. DsRNA-expressing *E. coli* caused a concentration-dependent loss of EGFP fluorescence (Figs. 2A, B). The effectiveness of 50 µl of dsRNA-expressing *E. coli* (approximately 5×10^7^ bacterial cells, or 10 per S2 cell) was comparable to that of 1 µg of naked dsRNA (Fig. 2B). To test the specificity of *E. coli*-mediated RNAi, we challenged S2 cells with bacteria that expressed dsRNA for either amphiphysin or syndapin, proteins sharing a degree of structural similarity and containing both BAR and SH3 domains. On knockdown, syndapin levels dropped to as low as 3% of those in control cultures and amphiphysin was undetectable on Western blots. Knockdown of either gene product did not affect the protein levels of the other, showing that RNAi was specific to the gene product targeted (Fig. 2C, D). Identical amounts of bacteria produced different levels of protein knockdown for each gene (Fig. 2C, D), but this may reflect different rates of protein turnover or different amounts of dsRNA produced from each plasmid construct. In summary, dsRNA delivered in *E. coli* induces robust and specific RNAi in *Drosophila* S2 cells.

### Evidence in support of phagocytosis as a route of dsRNA entry

**Figure 3:**
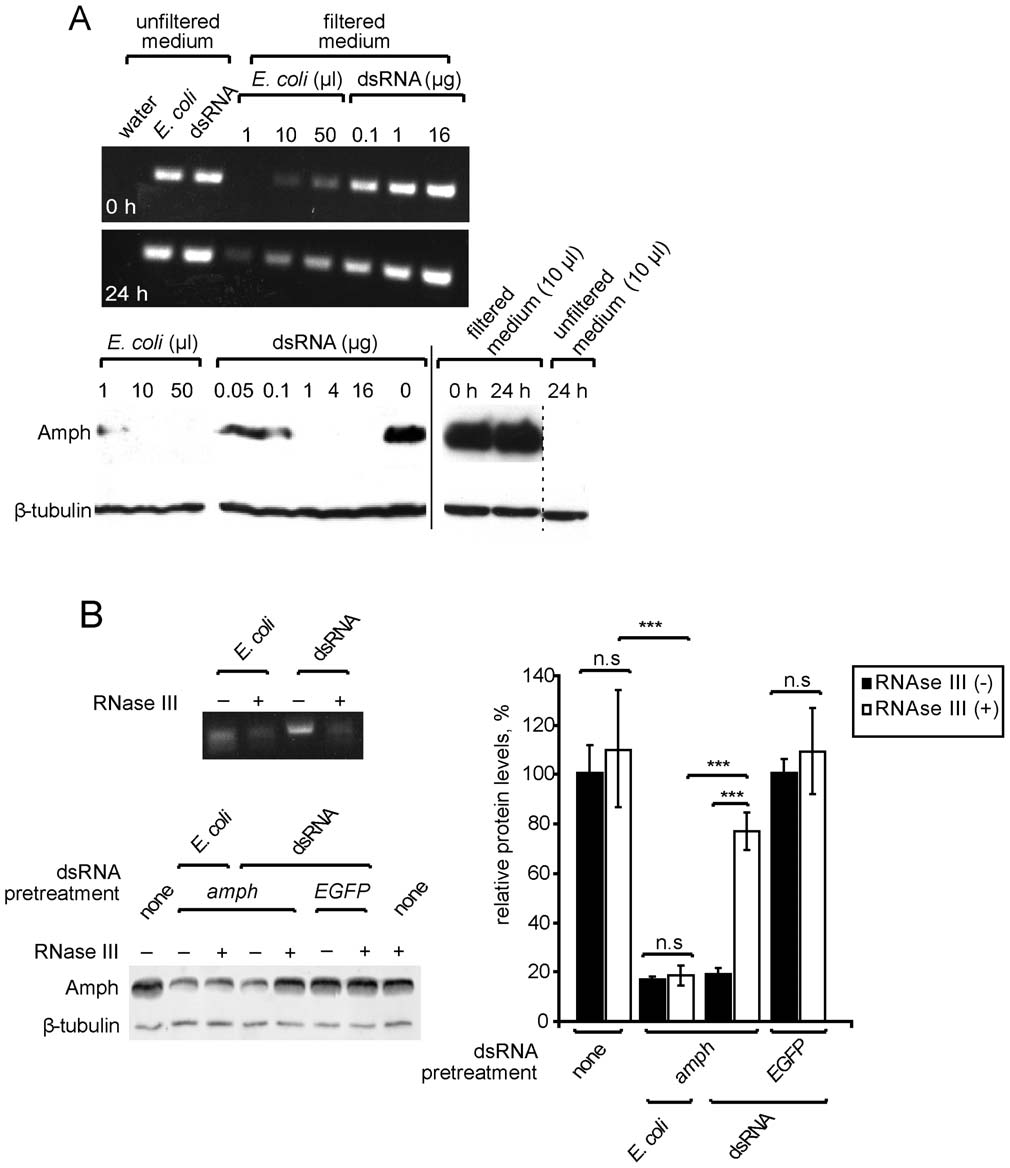
Free dsRNA cannot account for RNAi mediated by *E. coli*. (**A**) dsRNA leaking into the culture medium from *E. coli* that express dsRNA does not account for RNAi. Top: RT-PCR detection of *amph* dsRNA in cell culture medium incubated with either dsRNA-expressing *E. coli* or naked dsRNA, either unfiltered, or filtered through a 0.22 µm filter to remove bacterial cells. Samples were taken immediately (0 h) and 24 hours after *E. coli* or dsRNA was added. Bottom: Western blots showing amphiphysin and β-tubulin expression in S2 cultures incubated for 72 hours with varying amounts of *amph* dsRNA-expressing *E. coli*, naked *amph* dsRNA, or treated with 10 µl of unfiltered or filtered cell culture medium recovered from cultures incubated for 24 hours with *amph* dsRNA-expressing *E. coli*. (**B**) RNAi mediated by *E. coli* is RNase III insensitive. Top left: RT-PCR detection of *amph* dsRNA in filtrates of cell culture medium incubated with either dsRNA-expressing *E. coli* or naked dsRNA, treated (+) or non-treated (−) with RNase III. Samples were taken 24 hours after *E. coli* or dsRNA was added. Bottom left: Western blots showing amphiphysin protein levels in S2 cultures incubated with *amph* or *EGFP* dsRNA, or *E. coli* expressing *amph* dsRNA, treated (+) or non-treated with RNase III (−). Right: bar graph shows protein levels of amphiphysin relative to β-tubulin. Values are averages of four independent experiments. Error bars indicate standard error of the mean. *** P<0.005, n.s. P>0.05; two-tailed t-test.

We next tested whether bacterially mediated RNAi could be explained by dsRNA leakage from bacteria. We treated S2 cells with either naked *amph* dsRNA or *E. coli* that expressed *amph* dsRNA, and removed all cells by filtration at two time-points. We detected dsRNA in filtered medium from cultures incubated with bacteria for 24 hours, but not enough to explain a strong RNAi effect (Fig. 3A). For example, 10 µl of dsRNA-expressing *E. coli* robustly knocked down amphiphysin but filtered medium from the same culture did not, and this volume of filtered medium contained less than 0.1 µg of naked *amph* dsRNA, which only weakly knocked down amphiphysin. Furthermore, filtrates of cell culture medium in which dsRNA-expressing bacteria had been incubated failed to knock down amphiphysin (Fig. 3A). To exclude the possibility that a small but continuous leakage of dsRNA from bacteria contributes to RNAi we tested whether RNAi induced by bacteria is RNase III-sensitive. We pre-treated naked dsRNA and bacteria that expressed *amph* dsRNA with RNAse III, and carried out RNAi in the presence of RNase III; RNase treatment was efficient because the levels of dsRNA detected in filtrates of cell culture media after 24 hours were significantly reduced (Fig. 3B). More importantly, levels of amphiphysin dropped by over 80% after RNAi treatment with bacteria, irrespective of whether bacteria had been RNase III treated or not (Fig. 3B). In contrast, RNase III treatment inhibited dsRNA-induced RNAi (Fig. 3B). Therefore bacterially-induced RNAi is insensitive to RNase III treatment. Together these results indicate that free dsRNA cannot account for RNAi induced by bacteria.

**Figure 4:**
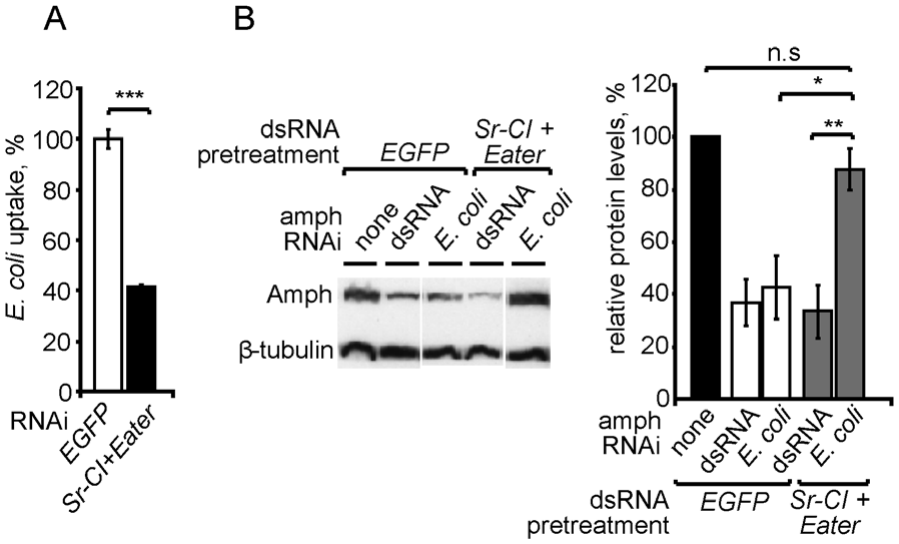
Dependence of *E. coli*-mediated RNAi on Sr-CI and Eater. (**A**) Inhibition of phagocytosis of *E. coli* after RNAi treatment for either EGFP (control) or Sr-CI and Eater. (**B**) Inhibition of phagocytosis impairs RNAi mediated by dsRNA-expressing *E. coli* but not by naked dsRNA. Western blot for Amph and β-tubulin from cultures of S2 cells pretreated with RNAi for either EGFP (control) or Sr-CI and Eater, and subsequently treated with either naked *amph* dsRNA or *E. coli* that express *amph* dsRNA. Bar graph shows levels of amphiphysin protein relative to β-tubulin. In all panels, error bars indicate standard error of the mean. * P<0.05; ** P<0.01; *** P<0.005; n.s. P>0.05; two-tailed t-test. All quantitative data are based on at least three independent experiments.

We also tried to distinguish between endocytic or phagocytic uptake routes for dsRNA in *E. coli*-mediated RNAi. Uptake of free dsRNA in *Drosophila* S2 cells requires clathrin-mediated endocytosis [7], [8], and indeed clathrin knockdown inhibited subsequent knockdown of amphiphysin (Fig. S1A). However, knockdown of clathrin also blocked phagocytic uptake of *E. coli* (Fig. S1B) and therefore does not distinguish between endocytic and phagocytic mechanisms of RNAi. Similarly, depolymerisation of actin by cytochalasin D, previously shown to impair phagocytosis of *E. coli* in S2 cells [11], also inhibited clathrin-mediated internalisation of naked dsRNA (Fig. S2) and could also not distinguish between endocytic and phagocytic uptake. However, knockdown of scavenger receptors Eater and Sr-CI was previously reported to inhibit phagocytosis of *E. coli* [12], [13], but not RNAi mediated by naked dsRNA in S2 cells [7], [8]. In agreement with previous reports, simultaneous knockdown of Eater and SR-CI inhibited phagocytosis (Fig. 4A) but not RNAi by naked dsRNA (Fig. 4B). It also inhibited *E. coli*-mediated RNAi (Fig. 4B), supporting the delivery of dsRNA by Eater/SR-CI-mediated phagocytosis of *E. coli* rather than endocytic uptake of naked dsRNA.

In conclusion, S2 cells can take up dsRNA by a phagocytic route that is independent of naked dsRNA uptake, and this pathway of dsRNA entry is highly effective in RNAi. Although RNAi in *C. elegans* can also be mediated by ingestion of dsRNA-expressing bacteria [14], digestion of these in the gut is likely to release naked dsRNA, which is a substrate for the SID-1 uptake channel [15]. Our results argue that systemic spread of RNAi in animals does not absolutely require naked dsRNA, but could occur via phagocytosis of dsRNA-containing microorganisms, apoptotic cells, or cell fragments such as exosomes. In *Drosophila* systemic RNAi is essential for antiviral immunity [16], and phagocytosis of virus-infected cells or cell fragments could be a mechanism for this. The same pathway could also be exploited by pathogens as a potential route to target professional phagocytes by RNAi. We also speculate that phagocytic spread of RNAi might have physiological consequences in other species, including mammals, mediated by different types of phagocytic cells such as macrophages, dendritic cells and glia.

## Methods

### Cell culture, RNAi and RNase III treatment, microscopy and immunostaining

S2 cells were maintained as described [17]. Unless otherwise indicated, RNAi was performed for 72 hours in 6-well plates, using 1–16 µg of dsRNA and 1×10^6^ S2 cells (cell density confirmed by hematocrit counting) in 1 ml of complete medium per well. Before addition of dsRNA to the cell culture media cells were pre-incubated for 1 hour in serum free medium. RNase III (Cambio, UK) pre-treatment of either naked dsRNA or bacteria expressing dsRNA was carried out at 37C for 1 hour using 5 units of RNase III. After addition of naked dsRNA or bacteria to the S2 cell cultures RNase III (2 units) was added to the cell culture media. To generate a stable cell line expressing EGFP-Rab5, pMTBlast-EGFP-Rab5 was constructed from a 1.5 kb *Not*I-*Xba*I EGFP-Rab5 from UAS-GFP-Rab5 [18] ligated to pMTBlast; this vector derives from pIB/V5-His Topo (Invitrogen, Paisley, UK), in which we replaced a 567 bp *Bsp*HI*/Kpn*I fragment carrying the constitutive Opie-2 promoter with a 616 bp *Kpn*I/*Sfo*I fragment carrying the inducible methallothionein promoter (P_mt_), from pMT/V5-His-A (Invitrogen, Paisley, UK). S2 cells were transfected with 1 µg of pMTBlast-EGFP-Rab5 using Cellfectin (Invitrogen, Paisley, UK) and transfectants were selected with blasticidin. Blasticidin-resistant transfectants were sorted by fluorescence activated cell sorting (FACS) for mild EGFP expression and cultured thereafter in complete medium supplemented with 10 µg/ml of blasticidin. Expression of EGFP-Rab5 was induced with 1 mM CuSO_4_ for 4 hours prior to fixation, when required. Cells processed for immunostaining were treated as described [17]. Rabbit anti-Sticky [10] was used at 1∶500 and rat anti-tubulin (clone YL1/2; Sigma-Aldrich) was used at 1∶40 dilution. Secondary antibodies were labelled with FITC (1∶200, Jackson ImmunoResearch Inc., West Grove, PA) or Alexa Fluor 594 (1∶200, Molecular Probes, Eugene, OR). Nuclear DNA was stained with 1 µg/ml Hoechst-33342 (Invitrogen, Paisley, UK) in PBS for 10 min. Cells were viewed in an MRC BioRad 1024 confocal microscope mounted on a Nikon Eclipse E800 microscope. Images were captured using a 60×/NA1.4 objective, and exported using LaserSharp software (BioRad, Hemel Hempstead, UK). Brightness, contrast, levels and color channels were adjusted using Photoshop (AdobeSystems, San Jose, CA).

### RNA synthesis and RT-PCR

DNA templates for transcription by T7 RNA polymerase were amplified by PCR as follows. Eater, Sr-CI, clathrin heavy chain (CHC) and Syndapin were amplified from genomic DNA template using primers (MWG, Ebersberg, Germany) as specified (www.dkfz.de/signaling2/e-rnai/); Amphiphysin, cDNA clone LD19810 using primers taatacgactcactataggt GGAGCGGTTACGATGCCC and taatacgactcactataggt GTGGCCAATTTGTCGACGA; EGFP, pMTBlast-EGFP-Rab5 with primers taatacgactcactataggt ATGGTGAGCAAGGGCGAGGAGCT and taatacgactcactataggt CTTGTACAGCTCGTCCATGCCGAG (T7 promoter sequences are underlined). Synthesis of dsRNA and RT-PCR were carried out as described [17]. Amphiphysin primers used in RT-PCR experiments were: TGGACAGTTTCCCGAAATGAAGA and GCTCGTCATGCAGTTCCGTGTTC, which amplify an intervening intronic sequence from genomic template.

### Bacterial expression of dsRNA and bacteria-mediated RNAi

dsRNAs were synthesized in RNaseIII-deficient *E. coli* HT115, using IPTG-inducible T7 RNA polymerase and pPD129.36 plasmid vector [9]. All gene fragments were sequenced in both strands: a 0.5 kb *Bgl*II fragment of *sticky* cDNA clone RE26327 [10], a 0.6 kb *Bam*HI/*Hind*III fragment of pGEX-SyndN (see syndapin antibodies), and nucleotides 354 to 1120 of *amph* cDNA clone LD19810 [19] generated by PCR using primers ggatcccaacga ATGACCGAAAATAAAGGCATAATGCTGG and cccggg GATGGGATTGCTTGTTTGCTTGCGTAGCGT (henceforth, gene sequences in primers are in uppercase and restriction sites are underlined). Nucleotides 1–717 of EGFP (pEGFP Sequence, www.addgene.org) were amplified from pMTBlast-EGFP-Rab5 (see below) using primers ggggtacccacc ATGGTGAGCAAGGGCGAGG and ggaattccatatgt TTGTACAGCTCGTCC.

A single *E. coli* HT115 colony harbouring each plasmid construct was grown overnight at 37°C in liquid culture with shaking. An inoculum of this culture was diluted 1∶500 to start a fresh culture next morning. At OD_600_ 0.4–0.6 this was treated with 1 mM IPTG for 2 hours. Bacteria were settled by centrifugation for 1 min at 100 g and resuspended in *Drosophila* Serum-Free Medium (Invitrogen, Paisley, UK) to OD_600_ of 1.2. Different volumes of the *E. coli* suspension, from 1–50 µl, were used to challenge a culture of 5×10^6^ S2 cells in complete medium, incubated at 25°C. OD_600_ of 1 corresponds approximately to 10^9^ bacterial cells per ml [20].

FITC-labelled *E. coli* (Molecular Probes, Eugene, OR) phagocytosis by S2 cells was performed essentially as described [13]. We used a Becton Dickinson FACScan and a Becton Dickinson LSR to count 10,000–20,000 cells from each sample. Results were analysed using Summit (DakoCytomation, Fort Collins, CO). At least three independent experiments were performed for each condition.

### Western blots and syndapin antibodies

S2 extracts were prepared as described [17]. Blots were probed with rabbit anti-syndapin 1∶1000, rabbit anti-amphiphysin 1∶2000 [19], or mouse anti-alpha-tubulin 1∶3000 (clone E7, Developmental Studies Hybridoma Bank, http://dshb.biology.uiowa.edu) and developed on film by Enhanced Chemiluminescence (ECL), except blots carried out regarding RNase III treatment (Fig. 3B). These were developed using IRDye® secondary antibodies and imaged on an Odyssey® Fc imaging system (Li-Cor Biotechnology, UK). In all cases band densities on blots were quantified from 3–4 independent experiments. To generate an N-terminal fragment of syndapin (codons 5–212, sequence Q9VDI1) fused to glutathione-S-transferase (GST), a PCR product was amplified from cDNA clone LD46328 (Geneservices, Cambridge, UK) using primers: 5′-aagaattcg CAGCGATGATCAGCTCCTC-3′ (sense) and 5′-tgatgaagctttta TTCGGCAATCGCCTGCTC-3′ (anti-sense), digested with *Eco*RI and *Hin*dIII, cloned in pGEX-KG [21] to generate pGEX-SyndN, and sequenced on both strands. Recombinant protein was purified from BL21(DE3) *E. coli* (Novagen, UK) on glutathione immobilised Sepharose 4B beads, and digested with thrombin according to the manufacturer's instructions (GE Healthcare, UK) to release the syndapin fragment. Antibodies were generated against this fragment in rabbits by Novagen (UK).

## Supporting Information

Figure S1 Inhibition of clathrin-mediated endocytosis inhibits both *E. coli*-mediated RNAi (**A**), and *E. coli* uptake in S2 cells (**B**). (**A**) Clathrin heavy chain (CHC) RNAi inhibits subsequent knockdown of amphiphysin using *E. coli* expressing *amph* dsRNA. Western blot showing amphiphysin protein levels in S2 cultures pretreated with *EGFP* (control) or *amph* dsRNA and subsequently incubated with *E. coli* that express either *amph* dsRNA or a control dsRNA synthesised from plasmid vector only. (**B**) Inhibition of *E. coli* uptake after RNAi treatment for either EGFP (control) or CHC.
(PDF)

Figure S2 Cytochalasin D (CytoD) treatment of *Drosophila* S2 cells inhibits both dsRNA-mediated and *E. coli* mediated RNAi. Western blots showing amphiphysin and β-tubulin protein levels in S2 cultures treated with CytoD or ethanol (EtOH, control) prior to Amph knockdown by RNAi using varying amounts of either naked *amph* dsRNA or *E. coli* that expressed *amph* dsRNA.
(PDF)
