# Supplementary figures and images for: A Phagocytic Route for Uptake of Double-Stranded RNA in RNAi
Source: PLoS One. 2011 Apr 29;6(4):e19087. doi: 10.1371/journal.pone.0019087 (PMC3084738; doi:10.1371/journal.pone.0019087)

A

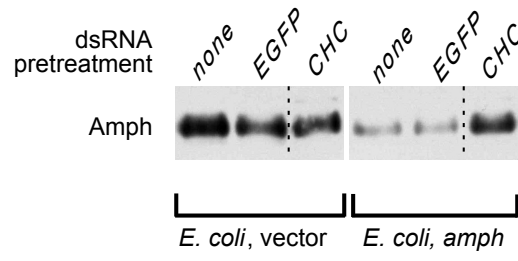

B

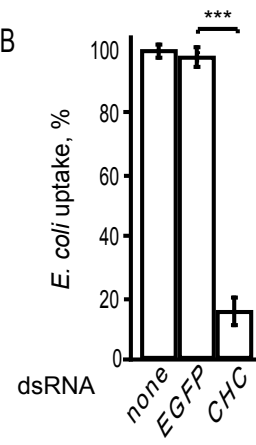

Supplement: Figure S1 — Inhibition of clathrin-mediated endocytosis inhibits both E. coli-mediated RNAi (A), and E. coli uptake in S2 cells (B). (A) Clathrin heavy chain (CHC) RNAi inhibits subsequent knockdown of amphiphysin using E. coli expressing amph dsRNA. Western blot showing amphiphysin protein levels in S2 cultures pretreated with EGFP (control) or amph dsRNA and subsequently incubated with E. coli that express either amph dsRNA or a control dsRNA synthesised from plasmid vector only. (B) Inhibition of E. coli uptake after RNAi treatment for either EGFP (control) or CHC. (PDF) [file pone.0019087.s001.pdf]

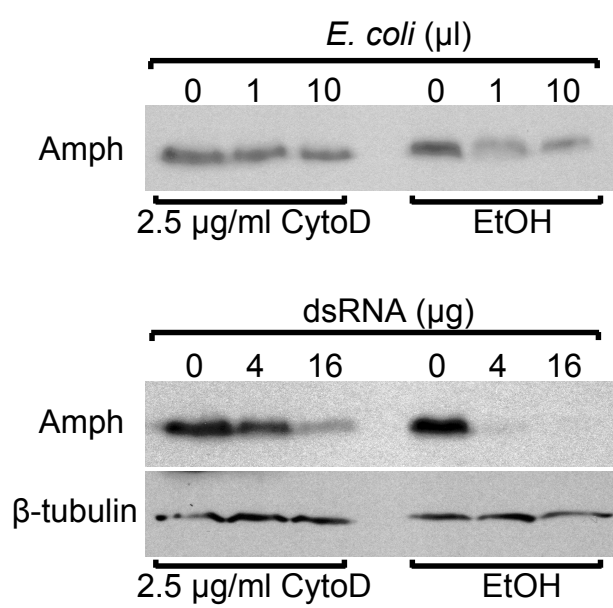

Supplement: Figure S2 — Cytochalasin D (CytoD) treatment of Drosophila S2 cells inhibits both dsRNA-mediated and E. coli mediated RNAi. Western blots showing amphiphysin and β-tubulin protein levels in S2 cultures treated with CytoD or ethanol (EtOH, control) prior to Amph knockdown by RNAi using varying amounts of either naked amph dsRNA or E. coli that expressed amph dsRNA. (PDF) [file pone.0019087.s002.pdf]
